# Supplementary material for: Use of “The Knowledge-to-Action Framework” for the implementation of evidence-based nursing in child and family care: Study protocol
Source: PLoS One. 2023 Mar 31;18(3):e0283656. doi: 10.1371/journal.pone.0283656 (PMC10065232; doi:10.1371/journal.pone.0283656)
Supplement: Attachment — Submitted filename: Reviewer x response .docx [file pone.0283656.s001.docx]

I was very interested and enjoyed while I am reading your research protocol. However, there were some points need to be considered if you would like to publish this peace of paper.

1. **Background:** Although your explanation about the structure of Knowledge-to-Action framework principles and dimensions is great, it would be lovely to add paragraph explaining the rational for your study and why other literature did not cover this area.
2. **Sample:** Please write more details about recruitment process and how you avoided bias while collecting your data.
3. **Data collection**: In research design section (under phase 1), you mentioned some research instruments, but you haven’t explained if they are valid or psychometrically evaluated. Please add a validity and reliability score for each.
4. **Discussion**: You need to add a section containing the expected contribution of the study to practice, policy and future research.
5. You need also to add conclusion at the end addressing all key findings together in a good manner.
