# Supplementary material for: Use of “The Knowledge-to-Action Framework” for the implementation of evidence-based nursing in child and family care: Study protocol
Source: PLoS One. 2023 Mar 31;18(3):e0283656. doi: 10.1371/journal.pone.0283656 (PMC10065232; doi:10.1371/journal.pone.0283656)
Supplement: Attachment — Submitted filename: Reponse reviewer.docx [file pone.0283656.s002.docx]

Cânia Torres

e-mail: caniabasto@gmail.com

Editor-in-Chef

PLOS ONE

Dear Editor-in-Chef,

Please find attached the revised version of the manuscript entitled “*Use of The Knowledge-to-Action Framework for the implementation of evidence-based nursing in child and family care: study protocol”*. All modifications were made in accordance to reviewers’ considerations and the answers to their comments are also attached. In our opinion significant modifications were carried out across the manuscript. The entire manuscript was revised by an English native speaker.

Thanks for your attention.

Yours sincerely,

Cânia Torres

**Answer to Reviewer 1**

We would like to thank for the reviewer effort and dedicated time to evaluate our manuscript.

**Point 1:**

The reviewer suggested *“For that reason, I believe that the authors of this manuscript is too ambitious in presenting the KTA Framework as well as the protocol for a future study for which the model is foundational. I believe this manuscript needs to be a stand-alone review paper which describes in much greater detail the process of developing the KTA Framework. (…)”*

**Response 1:** We are grateful for the suggestion, but carrying out an isolated review is not foreseen within the scope of the study protocol.

**Answer to** **Reviewer 2**

We would like to thank the reviewer effort and dedicated time to evaluate our manuscript. The points addressed allowed to improve considerably our work.

**Point 1:**

Background: Although your explanation about the structure of Knowledge-to-Action framework principles and dimensions is great, it would be lovely to add paragraph explaining the rational for your study and why other literature did not cover this area.

**Response 1:** A new point has been introduced in the document: Pertinence of the study.

*“With this protocol it is intended to build a conceptual and methodological basis for the study, based on the KTA model. The translation of knowledge in nursing practice is currently a focus for professional practice in family nursing and several studies evidence that this practice has very significant contributions in improving care. ^(25,26)^ The inclusion of stakeholders in all crucial stages of the research process will guarantee that it will be of interest to all the providers of nursing care to the child and family. With the involvement of pediatric nurses and parents/caregivers of hospitalized children, it is expected to evaluate the real contribution that the implementation of KTA model will have in care, from the perspective of all involved. Although in Portugal there is no study in the literature that uses KTA model in this context, in other countries the reality is different. This study provides nurses with a concrete strategy that allows structuring the process of professional practice of nurses, as regulated in the common competencies of nurses in the light of the National Nursing Association (Ordem dos Enfermeiros)”* (line 126 to 140).

**Point 2:**

Sample: Please write more details about recruitment process and how you avoided bias while collecting your data.

# Response 2: At the point of the Study Sample, it was added *“It should be noted that we considered that some sample could happen and, to safeguard them, we indicate some considerations. All nurses who are working in the service where the study will take place will be eligible to participate, however, the nurses participating in Phase I of the study will be the same as those who will be able to integrate Phases II and III. If new nurses are integrated into the service during the process, they will not be considered for the sample, but may, in the future and after the end of the study, be integrated. Nurses who, maintaining a bond with the institution, are absent from the service in phases II and/or III for personal reasons, may continue to participate in the study if they wish so”* (line 254 to 261).

**Point 3:**

Data collection: In research design section (under phase 1), you mentioned some research instruments, but you haven’t explained if they are valid or psychometrically evaluated. Please add a validity and reliability score for each.

**Response 3:** At the point of the Research Design - Phase 1: Identifying the Problem, “*The questionnaires that are expected to be used at this stage of the study all have psychometric validation for the Portuguese population, thus guaranteeing validity and reliability. The QABPBE-26 Portuguese version presents 26 items with an acceptable internal consistency (Cronbach's alpha =0.60). The Main Component Analysis suggests eight dimensions that explain 55.77% of total variance. The analysis conducted demonstrated valid empiric evidence and the questionnaire could be used in our context ^(18^). The QPSF presents an internal consistency of 0.94 ^(19)^. The ESCCE instrument for parents of hospitalized children ^(20)^, has adequate psychometric characteristics for the Portuguese population of parents of hospitalized children (Cronbach's alpha = 0.92), ensuring its reliability and validity for measuring satisfaction with nursing care"* (line 296 to 305).

**Point 4:**

Discussion: You need to add a section containing the expected contribution of the study to practice, policy and future research.

**Response 4:** A point was added that refers to the contributions of the study. *“This study may bring important contributions to the practice of nurses, health organizations and future studies. Its relevance in innovation and continuous improvement of pediatric nursing care is considerable because pediatric nurses are sought to integrate evidence into their professional practice. With the empowerment of nurses, through the training and monitoring of mentors in EBP, it will allow to produce evidence directed to the real context. In the literature, it is perceived that the use of the best evidence in the practice of nurses is a challenge, and, in this project,	we intend to develop strategies that potentiate the implementation of this evidence. Through the KTA model, and its structure, it will be possible to develop and sustain an environment that facilitates the translation of knowledge, specifically directed to the reality experienced, involving stakeholders: nurses, parents/caregivers in the crucial phases of the study. Also implies the development of nursing resources in the institution. With the EBP training that this study provides to nurses, it will allow them to develop competencies in clinical research and health services. Thus, the health organization will have more qualified human capital to carry out research. At the level of clinical governance there will be an impact, because it promotes the reduction of the variation of the practice of nurses and consequent increase in effectiveness. This study seems to offer support for the development of future research related to the use of KTA model or other, for the production, synthesis, and implementation of evidence in the practice of hospital nurses, seeking to contribute to the increase of scientific production in this theme, still challenging for nursing in Portugal"* (line 421 to 442).

**Point 5:**

You need also to add conclusion at the end addressing all key findings together in a good manner.

**Response 5:** A conclusion was included in the document. *“The implementation of KTA model in a pediatric sane hospital unit will provide valuable strategies that will allow the implementation of evidence-based nursing in the care provided to children and families. It will allow nurses to add scientific knowledge to their skills in caring for sick children and parents/caregivers, involving the family and their satisfaction with nursing care in this process. The assessment of the satisfaction of parents/caregivers before and after the implementation of the KTA model will be extremely relevant and will be fundamental for the definition of existing problems. In this study, the involvement of stakeholders, becoming partners in this process, has the potential to motivate the very conduct of the study, since it is expected a very positive influence, both in the development of competencies in EBP in nurses, and the improvement of the satisfaction of parents/caregivers with the care provided in the hospital unit. We hope with the implementation of KTA model, positively influence the view of pediatric nurses, on the use of scientific evidence in care. The implementation of this study will effectively be a challenge with an impact in pediatric nursing practice”* (445 to 460).
